# Epiphytic Plants: Perspective on Their Diversity, Distribution, Systematics and Conservation in the Changing Environment

**DOI:** 10.3390/plants14152265

**Published:** 2025-07-23

**Authors:** Thorsten Krömer, Sven Peter Batke

**Affiliations:** 1 Centro de Investigaciones Tropicales, Universidad Veracruzana, Xalapa 91000, Mexico; 2 Biology Department, Edge Hill University, Ormskirk L39 4QP, UK; sven.batke@edgehill.ac.uk

Epiphytic plants are vital components of tropical and subtropical forests, contributing significantly to biodiversity, ecosystem function, and structural complexity. Despite their importance, they remain understudied and face increasing threats from environmental change. This Special Issue, entitled “Epiphytic Plants: Perspective on Their Diversity, Distribution, Systematics and Conservation in the Changing Environment”, is a compilation of nine articles, whose authors deepen our understanding of the divergent roles of epiphytes, i.e., plants that germinate and root non-parasitically on other plants, mainly on trees. These are a conspicuous and integral component of tropical forest ecosystems in regard to local and regional plant diversity, especially in the Neotropics [1]. Roughly 10% of all vascular plants in the world are epiphytes, which are distributed in about 900 genera and 80 families. However, 85% of the more than 27,600 species recorded globally belong to only five taxonomic groups: Araceae, Bromeliaceae, Orchidaceae, *Peperomia* Ruiz & Pav., and Pteridophytes. Vascular epiphytes fulfill diverse ecological functions in tropical ecosystems, such as the accumulation of water and nutrients, in addition to amplifying biodiversity by providing microhabitats and food to different taxa (e.g., invertebrates, amphibians, reptiles, birds, and mammals). Due to their dependence on host trees and atmospheric water sources, epiphytes are considered one of the most endangered plant groups. Landscape fragmentation and forest degradation resulting from human activity are among the major threats to their survival.

This vulnerability is further illustrated by findings from a literature review by Krömer et al. (2025) [2], which showed that none of the human-modified ecosystems examined were able to fully replicate the epiphyte diversity found in undisturbed primary forests. Human disturbance in tropical forests typically leads to substantial diversity loss, reduced ecosystem services, and decreased soil health. However, over time, secondary forests may assist in the recovery of a substantial portion of species diversity and functional roles and complement primary forest remnants by enhancing landscape connectivity. Even so, primary forests are irreplaceable for epiphyte diversity conservation, and efforts to mitigate land-use impacts should focus on preserving these areas and implementing sustainable land-management practices.

Beyond secondary forests, urban environments represent an even more extreme form of habitat modification, where structural simplification and altered microclimates often lead to a further decline in epiphyte diversity. In a second paper by Landeros-López et al. (2025) [3], it was shown that the structural alterations of urban forests create stressful microclimatic conditions that can influence the morphology of sensitive plants, such as ferns. Sites with greater modifications in vegetation structure exhibited increased canopy openness, solar radiation, and temperature, but a lower relative humidity. Here, the fern leaves showed an increase in dry matter content and vein density, indicating a greater investment in resource storage and structural resistance. In the less-disturbed sites, terrestrial ferns demonstrated larger leaf area and specific leaf area, whereas epiphytes generally had smaller leaves, which could represent an adaptive advantage in more stressful environments.

These kinds of morphological shifts reflect broader ecological responses among epiphyte taxa to human disturbance. While certain patterns emerge, such as changes in abundance or adaptive traits, there is considerable variation across studies and species groups, cautioning against overly broad generalisations [2]. However, in a third paper, Siaz Torres et al. (2024) [4] found a higher abundance and species richness of epiphytic bromeliads in a disturbed forest than in a primary forest. Atmospheric species like *Tillandsia baileyi* Rose ex Small, *T. ionantha* Planch., and *T. usneoides* (L.) L. were most abundant on *Taxodium mucronatum* Ten., a dominant tree in the disturbed gallery forest. These bromeliads have morphological adaptations such as narrow leaves and abundant trichomes, which enhance their ability to capture atmospheric water and nutrients, facilitating their survival in disturbed or drier environments.

While some epiphyte species exhibit resilience in altered landscapes, broader environmental pressures continue to threaten the group as a whole. In addition to land-use change, the impacts of climate change, particularly rising temperatures and increased drought frequency, are emerging as major drivers of epiphyte decline. This is illustrated in a study on *Pleione formosana* Hayata, an endemic orchid once widespread across the mid-elevations of Taiwan, where Hsu et al. (2024) [5] found that populations have steadily declined, primarily due to orchid poaching and increasing climatic stress. Therefore, orchid plantlets were reintroduced to an old-growth cloud forest site in 2022, but the seedlings failed to survive the summer of 2023. The rising temperatures and frequent drought events threatened orchid growth, potentially increasing susceptibility to pathogens.

Alongside climatic stressors, direct human exploitation also poses a serious threat to many epiphyte populations. In particular, the mostly illegal harvest of species for commercial, ornamental, and cultural purposes has led to local declines across various taxa [2]. Due to their horticultural and ceremonial value, epiphytic orchids are often sold in local markets, but are also used in traditional medicine or as food supplements in tropical countries. *Prosthechea karwinskii* (Mart.) J.M.H. Shaw is an epiphytic orchid endemic to Mexico, threatened by the destruction of its habitat and the extraction of specimens to meet its demand for ornamental and religious use. Most of its populations are found in Oaxaca state, where variations in certain floral traits have been observed. A morphometric analysis by Santos-Escamilla et al. (2024) [6] identified the most significant floral characters as potential taxonomic markers for *P. karwinskii*, demonstrating the species’ value in linking morphological variation to geographic origin.

Taxonomic challenges associated with floral diversity are also evident in the orchid genus *Cymbidium* Sw., which displays intricate floral structures, strong endemicity, and a fragmented distribution. These features have led to a remarkable range of morphological forms, but they have also complicated efforts to classify the genus with consistency. To clarify the phylogenetic relationships within the genus *Cymbidium*, Peng et al. (2025) [7] used DNA barcoding and found a consistent separation between epiphytic and terrestrial species. Both genetic and morphological analyses supported this distinction, with epiphytic orchids forming a clearly defined clade.

Understanding the evolutionary relationships of epiphytes also requires examining other structurally dependent plant groups that share similar ecological strategies. Epiphytes are one of four such types, alongside climbing plants like lianas, nomadic vines, and hemiepiphytes, all of which rely on host structures for support at some point in their life cycle [1]. The latter have captured the attention of biologists since they seemingly hold clues to the evolution of epiphytes themselves. During fieldwork conducted in Papua New Guinea, Sundue and Maraia (2024) [8] documented seven hemiepiphytic species of ferns that all started growth as low-trunk epiphytes, and later, as larger climbing plants, exhibited long feeding roots that entered the soil. These new records expand the geographic and taxonomic breadth of hemiepiphytic ferns and offer morphological and phylogenetic clues to uncover additional records.

Studying structurally dependent plant types in a comparative way may produce important insights into possible positive or negative interactions between them. Thus, another study by Ceballos et al. (2025) [9] aimed to evaluate the co-occurrence of vascular epiphytes and lianas in northwestern Argentina. Both plant groups were found together on 20% of the sampled trees; they colonized the same canopy tree species with larger diameters, whereas smaller trees were typically colonized by either lianas or epiphytes, but not both. Epiphyte species were more likely to co-occur with liana species with specialized climbing mechanisms. Tree size and forest type (mature vs. successional) emerged as key factors influencing their co-occurrence.

Several studies on richness patterns of vascular epiphytes suggested that abundance and diversity are highest in humid montane forests at intermediate elevations [1]. Although some studies have analyzed epiphytes alongside other plant life forms, comparisons of elevational patterns in both epiphyte and tree diversity and biomass in tropical dry forests remain scarce. In southwestern Ecuador, Werner and Homeier (2024) [10] observed that tree species density and total species richness increased with elevation, while basal area and biomass showed no consistent trends. In contrast, epiphyte species density, richness, and biomass all increased markedly with elevation. The authors attribute these patterns, particularly for epiphytes, to rising humidity at higher elevations, reflecting increasingly mesic conditions.

Taken together, the contributions to this Special Issue offer a timely and comprehensive perspective on the ecology, evolution, and conservation of epiphytic plants. By spanning a wide range of taxa, habitats, and methodological approaches, these studies not only deepen our scientific understanding but also highlight the urgent need to protect this vulnerable and ecologically vital plant group. We hope this collection will serve as a valuable resource for researchers, educators, and conservation practitioners alike, and that it will help stimulate further work to explore, document, and conserve the remarkable diversity of epiphytes worldwide.
